# Cerebral Oxygenation and Autoregulation in Very Preterm Infants Developing IVH During the Transitional Period: A Pilot Study

**DOI:** 10.3389/fped.2020.00381

**Published:** 2020-07-15

**Authors:** Anna Giulia Cimatti, Silvia Martini, Silvia Galletti, Francesca Vitali, Arianna Aceti, Giulia Frabboni, Giacomo Faldella, Luigi Corvaglia

**Affiliations:** ^1^Neonatal Intensive Care Unit, S. Orsola-Malpighi University Hospital, Bologna, Italy; ^2^Department of Medical and Surgical Sciences (DIMEC), University of Bologna, Bologna, Italy

**Keywords:** IVH, NIRS, cerebral oxygenation, cerebral autoregulation, transitional period, echocardiography, preterm infants

## Abstract

**Background:** The transitional period, defined as the first 72 h after preterm birth, is often characterized by a significant hemodynamic instability, which represents an important risk factor for such neurological complications of prematurity as intraventricular hemorrhage (IVH). The impairment of cerebral autoregulation plays a key role in the pathogenesis of IVH, whose incidence is highest during the transitional period. This pilot study aimed to evaluate whether patterns of cerebral autoregulation and oxygenation differ in relation to IVH development in very preterm infants during the transitional period.

**Methods:** Infants <32 weeks' gestation were enrolled within 12 h from birth. A simultaneous monitoring of cerebral oxygenation (CrSO_2_) by near-infrared spectroscopy and of heart rate and peripheral oxygen saturation by pulse oximetry was performed over the first 72 h. Cerebral fractional oxygen extraction (cFTOE) and tissue oxygenation-heart rate reactivity index (TOHRx), which represents a marker of cerebrovascular reactivity, were calculated. Daily cranial and cardiac ultrasound scans were performed, in order to assess the hemodynamic status and to detect a possible IVH onset. CrSO_2_ and cFTOE, clustered on 6-hour epochs, were compared between infants who developed IVH during the study period and those who did not. A between-group comparison of TOHRx before and after IVH detection was also performed.

**Results:** Twenty preterm infants with a median gestational age of 27 weeks (interquartile range, IQR: 25-30 weeks) and median birth weight of 895 g (IQR: 822-1208 g) were enrolled. Of these, 8 developed IVH. The median age at IVH detection was 40 h (IQR: 30-48 h). Pre-IVH TOHRx was significantly higher compared to matched control periods (*p* <0.001). CrSO_2_ was significantly lower from 12 to 30 h and from 42 h onwards in cases compared to controls; however, a temporary CrSO_2_ rise preceded IVH detection. Similarly, cFTOE was significantly higher in IVH infants from 12 to 30 h and from 48 to 72 h, with a transient decrease between the two periods.

**Conclusions:** In preterm infants during the transitional period, the development of IVH is preceded by transient changes in cerebral oxygenation and oxygen extraction which, in turn, may underlie an early impairment of cerebral autoregulation. Larger studies are needed to confirm these preliminary findings.

## Background

Germinal matrix-intraventricular hemorrhage (GMH-IVH) is a common complication of premature birth, with a global estimated incidence of 35% among very preterm infants (1), and represents a possible risk factor for adverse neurodevelopmental outcome (2). Nearly 80-90% of GMH-IVH occur during the first 72 h of life (3, 4), which are characterized by the progressive hemodynamic transition from a fetal to a neonatal circulation. The *primum movens* in GMH-IVH development is a bleed in the sub-ependymal germinal matrix, which is rich in immature vessels poorly supported by the connective tissue (5). The hemorrhage may be limited to the germinal matrix region, or it may extend into the adjacent ventricular system; a parenchymal hemorrhage in combination with a GMH-IVH is referred to as periventricular hemorrhagic infarction.

The intrinsic fragility of the germinal matrix vasculature and cerebral blood flow disturbances are major contributors to the multifactorial etiology of GMH-IVH (6). Among other etiopathogenic mechanisms, the role for an early impairment of cerebral autoregulation, which leads to blood pressure-passive brain perfusion, has been reported (7, 8). Lower stroke volume and systemic blood flow before GMH-IVH occurrence have also been described (9, 10), thus suggesting a possible ischemia-reperfusion mechanism of injury. A better understanding of the early hemodynamic changes associated with GMH-IVH development would be helpful to reduce or prevent this complication. Near-infrared spectroscopy (NIRS) monitoring of cerebral oxygenation (CrSO_2_) provides useful information on neonatal brain haemodynamics (11). Moreover, the index of correlation between heart rate (HR) and CrSO_2_ (TOHRx) has also been proposed as a non-invasive marker of impaired cerebrovascular reactivity in preterm neonates during the transitional period (12).

This pilot study aimed at evaluating the patterns of CrSO_2_, cerebral fractional oxygen extraction (cFTOE) and TOHRx in relation to IVH development in very preterm infants in the first 72 h of life.

## Methods

Preterm infants born between June 2016 and December 2017 and admitted to the Neonatal Intensive Care Unit (NICU) of S. Orsola-Malpighi Hospital, Bologna (Italy) were consecutively enrolled in this prospective, pilot study within the first 12 h of life if they had a gestational age (GA) ≤32 weeks and a birth weight ≤1500 g. The presence of major congenital malformations, including congenital heart disease (CHD), was an exclusion criterion.

Informed consent to participate in the study was obtained from the parents/legal guardians of each infant. The study was conducted in conformity with principles and regulations of the Helsinki Declaration. The study protocol was approved by the Institutional Ethics Committee of S. Orsola-Malpighi Hospital, Bologna (Italy).

Infants were recruited within 12 h of life and underwent a continuous, simultaneous and synchronized monitoring of peripheral arterial oxygenation (SpO_2_), HR and CrSO_2_ up to 72 h of life. CrSO_2_ monitoring was performed using an INVOS 5100C oximeter (Covidien, Boulder, CO, USA), with the neonatal cerebral sensor placed on the forehead. SpO_2_ and HR were monitored using a Nellcor pulse oximeter (Covidien, Boulder, CO, USA), with the neonatal sensor placed preductally (right hand). NIRS and pulse oximeter traces were retrospectively analyzed using the ICM+ software (*https://icmplus.neurosurg.cam.ac.uk*, Cambridge Enterprise, UK), which includes a calculation engine that allows an easy estimation of complex parameters (13). Artifacts were identified and removed using tools included in software. Missing data (e.g., disconnections, sensor displacement periods, etc.) were also excluded from data analysis.

CFOE was calculated according to the following formula: [(SpO_2_-CrSO_2_)/SpO_2_] (14). CrSO_2_ and CFOE were averaged over 6-h periods, and the resulting values were used for statistical analysis.

**Figure 1:**
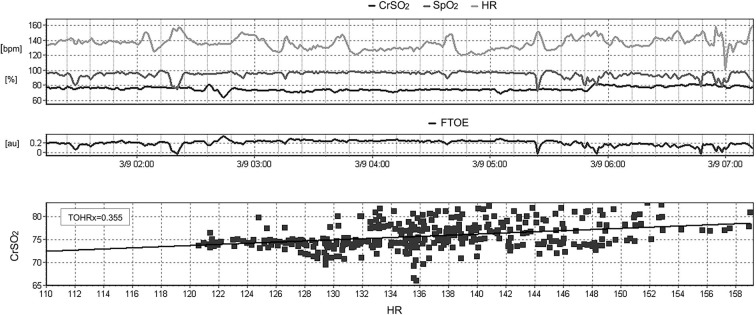
Example of the calculation of the moving correlation coefficient between heart rate (HR) and cerebral oxygenation (CrSO_2_), defined as TOHRx, over a 6-h period using the ICM+ software.

TOHRx was calculated over 6-h periods as the moving correlation coefficient between CrSO_2_ and HR using 5-min, 30-point epochs as previously described (12) (Figure 1). Time intervals with evidence of major artifacts, or with a missing data proportion >50% were excluded from the calculation. Positive TOHRx values were interpreted as markers of impaired autoregulation, whereas zero or negative values indicated intact autoregulation (12). For each infant, 2 periods were defined: one from the enrollment to GMH-IVH detection (pre), and the other from GMH-IVH detection to the end of the study monitoring (post). The median age at GMH-IVH development served as the time-splitting cut-off in the control group. TOHRx values, stratified into pre- and post-IVH periods in each study group, were thus included in the statistical analysis.

Ultrasounds were performed using a Philips HD11XE System (Philips Ultrasound, Andover, MA, USA); the first evaluation was carried out at the time of the study enrollment, and then repeated daily throughout the whole study period.

Cranial ultrasound scans (CrUSS), performed at 24-h intervals with a 8–5 Hz transducer through the anterior fontanel, were aimed to detect the presence of GMH-IVH as well as its localization (left/right) and severity, according to Volpe's grading (15).

Echocardiographic assessments were performed at 24-h intervals by a single expert operator, blinded to CrUSS findings, using a S12-4 Hz transducer. The presence of a patent ductus arteriosus (PDA) was evaluated from the suprasternal notch view combining two-dimensional and color-Doppler evaluations. Ductal diameter and trans-ductal Doppler pattern were examined, and the presence of a reverse flow in the abdominal aorta and in the anterior cerebral artery was also evaluated (16).

Left ventricular output (LVO) was calculated according to the formula [(left ventricular outflow velocity time integral [VTI]) x (HR) x (left ventricular outflow cross-sectional area)] and indexed to body weight (16). The left ventricular outflow diameter was measured from the parasternal long axis view using the leading-edge technique, whereas VTI was estimated from an apical five-chamber view with pulse-waved Doppler, sampling the left ventricular outflow tract. Angle correction was routinely used during the study echocardiograms in order to optimize LVO calculation.

Right ventricular output (RVO) was evaluated using a parasternal short axis view and calculated according to the formula [(right ventricular outflow VTI) x (HR) x (right ventricular outflow cross-sectional area)], indexed to body weight (17).

In the enrolled infants, the respiratory support during the study monitoring, surfactant administration, and daily levels of hemoglobin (Hb), partial arterial CO_2_ pressure (paCO_2_), systolic and diastolic blood pressure (measured non-invasively using the oscillometric method) were also recorded.

### Statistical Analysis

Data were analyzed using SPSS version 26.0 for Windows (Statistical Package for social Sciences, SPSS inc., Chicago, III, US). Data distribution was checked using Shapiro-Wilk test; since the data did not follow a normal distribution, non-parametric tests were used for statistical analysis. TOHRx values before and after IVH detection as well as daily clinical and hemodynamic parameters relevant to the study objectives were compared between GMH-IVH group and controls with Mann-Whitney U test. The impact of IVH on CrSO_2_ and cFTOE along with between-group differences in CrSO_2_ and cFTOE time trends were analyzed with a linear mixed-model regression, using an autoregressive repeated covariance type. Piecewise linear regression analysis, aimed at detecting points where a statistically significant change over time in the linear trend slope of CrSO_2_ and cFTOE occurred within each group was performed using the Joinpoint Regression Program, version 4.8.0.1 (18) as previously described (19). Statistical significance level was set at *p* < 0.05.

## Results

**Figure 2:**
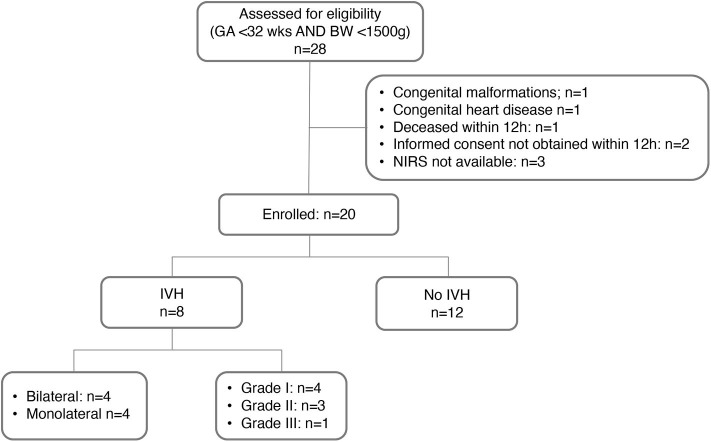
Flow-chart of the study phases. GA, gestational age; BW, birth weight; NIRS, near-infrared spectroscopy; IVH, intraventricular hemorrhage.

As documented in the study flow-chart (Figure 2), 20 preterm infants were included; in 8 out of 20 infants a GMH-IVH was detected at a median age of 40 h (interquartile range, IQR: 30-48 h). Details on GMH-IVH features are shown in Figure 2, whereas the main clinical characteristics of the study groups are summarized and compared in Table 1. Infants who developed GMH-IVH during the transitional period had a significantly lower GA and Apgar score at 5 min compared to those who did not. Moreover, the percentage of infants needing invasive respiratory support during the transitional period was significantly higher in the GMH-IVH group.

**Table 1:** Clinical characteristics of the study groups and results of between-group comparison.

**Neonatal characteristics**	**IVH (*n* = 8)**	**No IVH (*n* = 12)**	***P*-value**
Gestational age (weeks), median (IQR)	25.4 (25–26.7)	29.5 (27–32.3)	**0.031**
Birth weight (g), median (IQR)	844 (807–894)	1134 (830–1173)	0.069
Apgar Score at 5 min, median (IQR)	7 (5–9)	9 (9, 10)	**0.010**
Twins, *n* (%)	1 (12.5)	2 (16.6)	1.000
C-section, *n* (%)	7 (87.5)	11 (91.6)	0.653
Males, *n* (%)	7 (87.5)	7 (58.3)	0.187
Small for gestational age (SGA), *n* (%)	1 (12.5)	3 (25)	0.465
Antenatal steroids, complete course, *n* (%)	3 (37.5)	8 (66.6)	0.205
Mechanical ventilation, n (%)	6 (75)	1 (15.4)	**0.004**
Need for surfactant replacement, *n* (%)	7 (87.5)	6 (50)	0.106

*Bold indicates p-values < 0.05*.

The percentage of continuous data removed from the study analysis for technical reasons (e.g., artifacts, loss of signal, sensor displacement) as a function of hours of recording was 5.4% (IQR 4.2–6.4%).

Daily HR, PDA prevalence, Hb levels, paCO_2_, systolic and diastolic blood pressure, RVO and LVO in the two study groups are detailed in Table 2; no between-group difference was observed in these parameters.

**Table 2:** Clinical and hemodynamic variables of the two study groups during the transitional period and results of between-group comparison.

**Parameters**	**IVH (*n* = 8)**	**No IVH (*n* = 12)**	***P* value**
Heart rate (bpm), median (IQR)			
0-24 h	150 (144–157)	143 (137–150)	0.157
24-48 h	159 (150–162)	145 (134–167)	0.571
48-72 h	149 (143–161)	146 (130–159)	0.427
Hemoglobin (g/dl), median (IQR)			
0-24 h	16 (12.9–17)	16.3 (14.9–19.5)	0.385
24-48 h	14.2 (11–18.5)	16.9 (13.3–18.1)	0.462
48-72 h	12.5 (10.2–17.3)	16 (12.8–18)	0.216
PaCO_2_ (mmHg), median (IQR)			
0-24 h	42.7 (39.9–43.6)	40.7 (38.7–41.9)	0.157
24-48 h	43.9 (41–47.6)	41.3 (38–44.1)	0.473
48-72 h	42.1 (39.8–46.2)	40.4 (36.2–41.3)	0.208
Systolic blood pressure (mmHg), median (IQR)			
0-24 h	48 (44–56)	54 (47–58)	0.600
24-48 h	54 (50–60)	58 (51–62)	0.840
48-72 h	55 (49–63)	59 (57–63)	0.395
Diastolic blood pressure (mmHg), median (IQR)			
0-24 h	28 (22–35)	31 (25–33)	0.492
24-48 h	33 (29–40)	33 (29–45)	1.000
48-72 h	40 (36–47)	38 (36–40)	0.600
Patent ductus arteriosus, *n* (%)			
0-24 h	8 (100)	10 (83.3)	0.347
24-48 h	4 (50)	4 (33.3)	0.388
48-72 h	4 (50)	2 (16.7)	0.137
Left ventricular output (ml/kg/min), median (IQR)			
0-24 h	184.3 (153.7–184.3)	169.5 (135.7–246.8)	0.494
24-48 h	140.2 (133.7–237)	243.3 (176.3–341.6)	0.104
48-72 h	221.0 (174.3–409)	217.3 (164.3–289)	0.591
Right ventricular output (ml/kg/min), median (IQR)			
0-24 h	248 (199.2–335.8)	302.3 (263.6–540.3)	0.320
24-48 h	293.3 (237.2–425.7)	425.1 (234.7–582.2)	0.412
48-72 h	328.8 (228.5–448.6)	452.8 (395.5–475.4)	0.254

**Figure 3:**
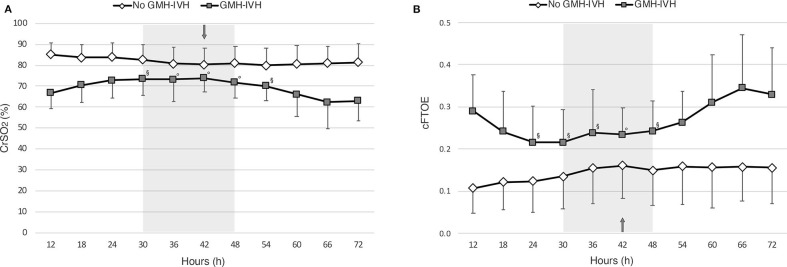
Patterns of cerebral oxygenation (CrSO_2_,) **(A)** and of cerebral fractional oxygen extraction (cFTOE) **(B)**, evaluated at 6-hour intervals over the first 72 h of life in infants who developed GMH-IVH (in gray) and those who did not (in white). The arrow indicates the median age at GMH-IVH detection, whereas the gray shadow represents the related interquartile time range. Linear mixed-model regression significances for time*IVH interaction are provided (^◦^*p* < 0.01; ^§^*p* < 0.05).

The linear mixed-model regression showed a significant effect of IVH (*p* < 0.001, *b* = −16.192), time (*p* = 0.026), and of the interaction between time and IVH (*p* = 0.020) on CrSO_2_. In particular, as shown in Figure 3A, CrSO_2_ time-trend patterns resulted significantly different between infants who developed GMH-IVH and those who did not from 30 to 54 h of life, which included the peri-IVH period. The within-group piecewise regression analysis of CrSO_2_ patterns documented a significant increase of this parameter (slope: 0.48, *p* < 0.05) between 12 and 36 h of life, followed by a significant decrease (slope:-0.53, *p* < 0.05) from 36 h onwards in the GMH-IVH group, whereas the control infants showed a slight CrSO_2_ reduction (slope:−0.19, *p* < 0.05) up to 42 h, followed by a plateau phase (slope: 0.03).

Similar findings were observed for cFTOE. In particular, a significant effect of IVH (*p* = 0.003, *b* = 0.141), time (*p* = 0.003) and of the interaction between time and IVH (*p* = 0.025) was observed on cFTOE, with significantly different time-trend patterns between the two study groups from 24 to 48 h of life (see Figure 3B). At the within-group piecewise regression analysis, a trend toward a cFTOE reduction (slope:−1.75) between 12 and 30 h, followed by a significant cFTOE increase (slope: 1.21, *p* < 0.05) up to 72 h of life was observed in GMH-IVH infants. In the control group, cFTOE gradually decreased (slope: 1.20, *p* < 0.05) from 12 to 42 h, and then outlined a plateau (slope: 0.09) until the end of the monitoring.

**Figure 4:**
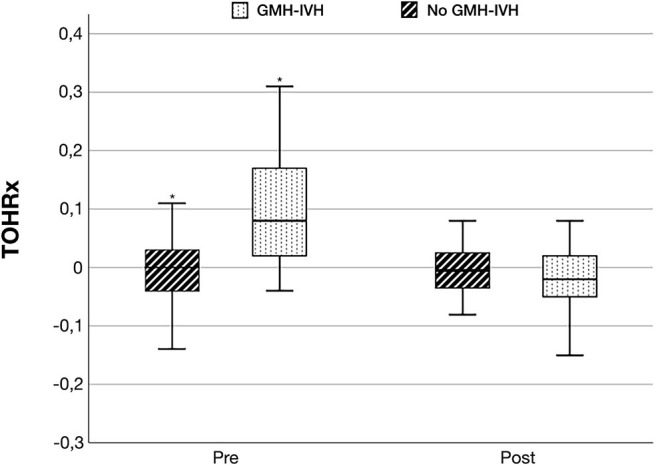
Tissue oxygenation-heart rate reactivity index (TOHRx) before and after GMH-IVH detection in infants who developed GMH-IVH and during matched time periods in the control group. *p < 0.001.

TOHRx values before and after IVH development (median value [IQR]: 0.08 [0.02-0.17] vs.−0.02 [−0.05, 0.02]), and during matched time periods for the control group (median [IQR]: 0.01 [−0.04, 0.03] vs.−0.002 [−0.03, 0.02]), are illustrated in Figure 4. Prior to GMH-IVH onset, TOHRx in case infants was significantly more positive compared to a matched time period in controls, whereas no difference between the two groups was observed in the subsequent phase.

## Discussion

According to the preliminary findings of this study, GMH-IVH onset during the transitional period may be associated with dynamic changes of cerebral oxygenation and oxygen extraction. Moreover, an early impairment of cerebral autoregulation, reflected by the more positive TOHRx values observed in the case group before IVH detection, may possibly underlie the development of this complication.

Due to the functional and anatomical immaturity of cerebral vasculature, preterm infants are at high risk of impaired cerebrovascular autoregulation, a physiological servomechanism aimed at protecting the brain from pressure-driven fluctuations of cerebral blood flow. The fragile periventricular vasculature that characterizes preterm neonates is particularly sensitive to the fluctuations of cerebral blood flow (CBF), thus placing these infants at high risk of GMH-IVH. An early identification of infants with impaired autoregulation would have a potential clinical benefit in order to limit potentially harmful CBF fluctuations. TOHRx is derived from the correlation coefficient between slow waves of HR and CrSO_2_ measured with NIRS, and has been previously proposed as an index of cerebral vascular reactivity in preterm neonates in the early post-natal period (12, 20) In particular, the authors reported more positive TOHRx values in infants with worse Clinical Risk Index for Babies II (12), which is an early neonatal risk index score for predicting morbidity and mortality; moreover, an increased passivity between CrSO_2_ and HR was also observed during arterial hypotension.

We observed significantly more positive TOHRx values before, but not after the development of GMH-IVH, compared to the control group during a time-matched period. We believe that this preliminary finding supports the possible pathogenic role of an impaired cerebrovascular autoregulation in GMH-IVH development (8, 21), and may also suggest a possible value for TOHRx in identifying preterm infants at higher risk for this complication during transition, although targeted studies on larger cohorts are needed.

GMH-IVH infants had a significantly lower GA and Apgar score compared to controls. This is consistent with previous data investigating the predictive risk factors for GMH-IVH development (22, 23). Although an inverse correlation between TOHRx and GA has been previously reported (12), in the present study the between-group difference was observed only before, and not after GMH-IVH development. Hence, we believe that this finding more likely reflects a transient impairment of cerebral autoregulation, rather than the effect of GA, which would be expected to be evident throughout the whole monitoring period. Furthermore, a significantly higher rate of infants who developed GMH-IVH were on mechanical ventilation; this data, which is consistent with previous evidence (24), may reflect a greater clinical instability in the GMH-IVH group. The present study, however, is not powered to evaluate the possible effects of mechanical ventilation on cerebral autoregulation and oxygenation.

Other NIRS-based correlation indexes have been proposed as autoregulation markers in animal and human studies. In particular, Brady et al. (25) analyzed the correlation between CrSO_2_ and cerebral perfusion pressure in a piglet model, identifying indices values >0.36 as a threshold for autoregulation loss, whereas Gilmore et al. (26) investigated the correlation index between CrSO_2_ and arterial blood pressure (ABP) in preterm neonates, adopting a cut-off value of >0.5 as an indicator of impaired autoregulation. Although slight methodological differences have to be acknowledged (i.e., adoption of HR rather than ABP or cerebral perfusion pressure for the correlation index calculation), we observed lower TOHRx values compared to the thresholds described for the aforementioned indexes. We may speculate that our data reflect a milder degree of autoregulation impairment, which could be supported by the vast predominance of low-grade IVH in our study population; nevertheless, further larger studies are required to investigate a possible correlation between TOHRx and IVH severity.

In the present study, significantly different patterns of CrSO_2_ and cFTOE were observed between case infants and controls, with particular reference to the peri-IVH period. While the trends of these parameter appeared relatively stable in the control group, GMH-IVH detection was preceded by a progressive CrSO_2_ increase and cFTOE reduction. These findings might suggest the occurrence of an early, transient phase of cerebral hyperperfusion in at-risk infants; in this context, a concomitant impairment of cerebral autoregulation may contribute to trigger IVH development. From the present data, however, it is not possible to evaluate whether the observed CrSO_2_ increase may underlie cardiovascular hemodynamic changes, as the echocardiography scans performed once daily may have been blind to the between-scan occurrence of hemodynamic fluctuations. Hence, this issue would deserve targeted investigations possibly using non-invasive continuous techniques for cardiac output monitoring (e.g., electrical velocimetry). Similar findings have been described by Noori et al., who investigated 12-h averaged CrSO_2_ and cFTOE patterns in a small cohort of extremely preterm neonates during transition (10). In particular, a CrSO_2_ rise and a simultaneous cFTOE decrease in the 12-h period prior to IVH onset was observed, whereas in the early monitoring phases and after IVH development case infants had lower CrSO_2_ and higher cFTOE compared to controls. The hypothesis of a relative cerebral hyperperfusion preceding IVH development has also been proposed by Alderliesten et al., who reported significantly higher CrSO_2_ and lower cFTOE values before, but not after the occurrence of a severe IVH in case infants compared to matched controls (8).

Verhagen et al. described a significant reduction of CrSO_2_ and increased cFTOE over the first week of life in preterm infants with GMH-IVH, the majority of which of low-grade (27). This data is consistent with the lower levels of CrSO_2_ observed in the IVH group compared to controls observed in the present study. However, the shorter monitoring period (i.e., 2 hours per day) adopted by Verhagen et al. from the second day onwards may have contributed to miss possible fluctuations in cerebral hemodynamic fluctuations associated with IVH development.

Changes in cardiac function in relation to IVH occurrence during the transitional period had been previously evaluated by Noori et al. (10), who observed that infants with IVH tended to have a lower LVO on the first day, followed by a trend toward an increase at 28 h. In the present study, no significant differences in daily LVO were observed between the groups throughout the study period. However, given the small number of infants on which our observation was based, and in consideration of the influence of the infant's ductal and hemodynamic status on this parameter, we believe that larger and targeted studies are needed to better understand the possible role of cardiac function changes on GMH-IVH development.

Targeted echocardiography represents the gold-standard technique for the assessment of cardiac function in preterm neonates; unlike NIRS, however, this technique does not allow a continuous hemodynamic evaluation, and may be blind to possible dynamic fluctuations occurring between the assessments. In this regard, combining functional echocardiography with non-invasive cardiac output monitoring techniques, which have been increasingly adopted in the neonatal population (28), may provide useful information.

In the present study, invasive ABP monitoring was not available; hence, the combined evaluation of other validated biomarkers of cerebral autoregulation, such as the correlation coefficient between CrSO_2_ and ABP, for which a normal cut-off is already available, was not technically feasible. A potential role of heart rate variability (HRV) in predicting impending IVH has been previously postulated (29). This parameter primarily reflects the infant's autonomic status and, as such, may be influenced by different factors (e.g., ventilation, inotropes, pharmacological ductal closure etc.) and is altered also in the context of other pathological conditions (e.g., sepsis, necrotizing enterocolitis) (30). HRV analysis, however, was not available for the present data; hence, whether the combination of HRV and TOHRx evaluation may improve IVH prediction has to be examined in targeted studies.

Cranial ultrasounds were performed daily, aiming to maintain a 24-h interval between the scans. This time interval, however, may have limited the accuracy of the timing of IVH detection, with possible implications on the interpretation of NIRS data.

The small study sample, together with the low number of high-grade IVH, also needs to be acknowledged among the study limitations. For this reason, a comparison of CrSO_2_ and cFTOE trend patterns between low-grade and high-grade IVH was not feasible, but deserves to be investigated in larger, targeted studies. Due to the pilot nature of this study, a priori sample size calculation was not performed; however, we believe that our preliminary findings may provide a potentially useful contribution to shed light on the complex pathophysiological mechanisms underlying GMH-IVH development.

In preterm infants during the transitional period, the development of IVH is preceded by a transient increase of cerebral oxygenation which, in turn, may result from an early impairment of cerebral autoregulation. Further studies on larger cohorts are needed to confirm these preliminary results and to evaluate the possible role of relevant clinical variables.

## Data Availability Statement

The raw data supporting the conclusion of this article will be made available by the authors upon reasonable request, without undue reservation.

## Ethics Statement

The study protocol was reviewed and approved by the Ethics Committee of S. Orsola-Malpighi Hospital. Written informed consent to participate in this study was provided by the participants' legal guardian/next of kin.

## Author Contributions

AC, SG, GFa, and LC designed the study. AC, SG, and FV enrolled the infants and collected the data. SM, AC, and AA performed data analysis. GFr contributed to the data analysis. AC and SM wrote the first draft.

## Conflict of Interest

The authors declare that the research was conducted in the absence of any commercial or financial relationships that could be construed as a potential conflict of interest.
